# Mitochondrial stress controls the radiosensitivity of the oxygen effect: Implications for radiotherapy

**DOI:** 10.18632/oncotarget.7412

**Published:** 2016-02-15

**Authors:** Richard B. Richardson, Mary-Ellen Harper

**Affiliations:** ^1^ Canadian Nuclear Laboratories (CNL), Radiobiology and Health, Chalk River Laboratories, Chalk River, ON, Canada; ^2^ McGill Medical Physics Unit, Cedars Cancer Center-Glen Site, Montreal, QC, Canada; ^3^ Department of Biochemistry Microbiology and Immunology, Faculty of Medicine, University of Ottawa, Ottawa, ON, Canada

**Keywords:** mitochondria, oxidative stress, oxygen effect, radiation therapy, therapy resistance

## Abstract

It has been more than 60 years since the discovery of the *oxygen effect* that empirically demonstrates the direct association between cell radiosensitivity and oxygen tension, important parameters in radiotherapy. Yet the mechanisms underlying this principal tenet of radiobiology are poorly understood. Better understanding of the oxygen effect may explain difficulty in eliminating hypoxic tumor cells, a major cause of regrowth after therapy. Our analysis utilizes the Howard-Flanders and Alper formula, which describes the relationship of radiosensitivity with oxygen tension. Here, we assign and qualitatively assess the relative contributions of two important mechanisms. The first mechanism involves the emission of reactive oxygen species from the mitochondrial electron transport chain, which increases with oxygen tension. The second mechanism is related to an energy and repair deficit, which increases with hypoxia. Following a radiation exposure, the uncoupling of the oxidative phosphorylation system (proton leak) in mitochondria lowers the emission of reactive oxygen species which has implications for fractionated radiotherapy, particularly of hypoxic tumors. Our analysis shows that, in oxygenated tumor and normal cells, mitochondria, rather than the nucleus, are the primary loci of radiotherapy effects, especially for low linear energy transfer radiation. Therefore, the oxygen effect can be explained by radiation-induced effects in mitochondria that generate reactive oxygen species, which in turn indirectly target nuclear DNA.

## INTRODUCTION

Many web sites of hospital radiology or radiotherapy departments state that nuclear DNA (nucDNA) is the most important radiosensitive target within cells, as it is involved in cell death, the initiation and therapy of cancer, and other biological effects. Supportive of the nucleus-centric view is the widely accepted premise that radiation-induced reproductive death of mammalian cells is principally due to lesions in nucDNA, particularly double-strand breaks (DSBs) [1]. These lesions are thought to lead to enhanced cell death in the presence of oxygen — the *oxygen effect* [2].

The best known explanation of the oxygen effect is the oxygen fixation hypothesis developed in the 1950s, which posited that radiation-induced non-restorable nucDNA lesions are lethal to cells in the presence of diatomic oxygen (O_2_) [3]. In the same decade, doubts arose that radiation damage was primarily genetic, based on studies that included, for example, the X-ray irradiation of giant amoebae, which showed that cell division is enhanced if these eukaryotes are fused with unirradiated mitochondria rather than nuclei [4]. Goldfeder [5] then proposed that mitochondria played an important role in determining cell radiosensitivity; this was based on the observation that cells highly endowed with mitochondria can function even if irradiation eliminates a substantial fraction of the mitochondria. Doubts have since emerged concerning the oxygen fixation hypothesis [3], not the least because the hypothesis fails to take into account nitric oxide (NO) as a radiosensitizer with similar effects as O_2_ (see Discussion).

**Figure 1:**
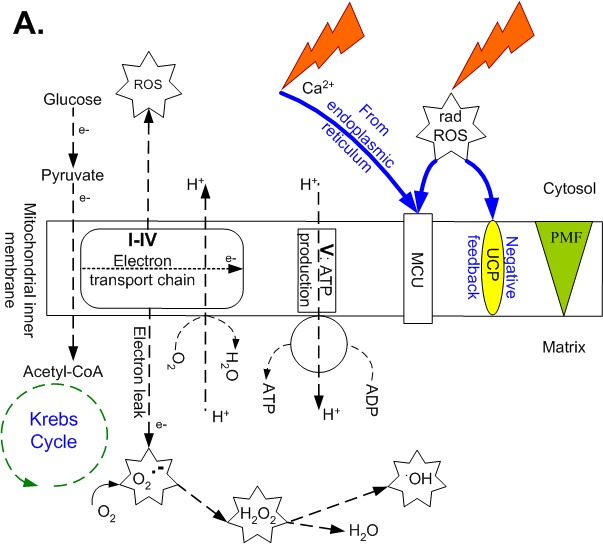

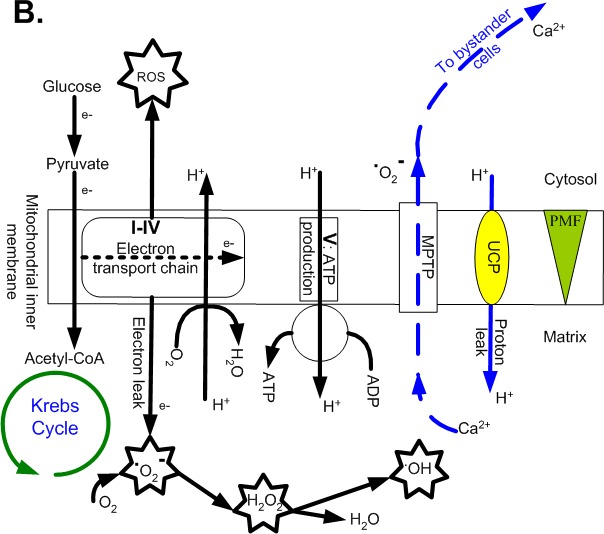
Ionic pathways across the mitochondrial inner membrane just before and after irradiation Pathways and components include the electron transport chain (complexes I-IV), ATP synthase (complex V), uncoupling proteins (UCP), mitochondrial Ca^2+^ uniporter (MCU), mitochondrial permeability transition pore (MPTP) and proton motive force (PMF, made up of membrane potential and the proton gradient). **A.** The moment just before ionizing radiation-induced ROS (radROS) and Ca^2+^ arrive at and cross the membranes (blue pathways), showing normal, coupled respiration at moderate levels of O_2_ uptake and ATP synthesis, accompanied by moderate electron leak and mtROS. **B.** The moment just after radROS and Ca^2+^ (at physiological levels) stimulate transient depolarization, ATP synthesis and O_2_ uptake, leading to electron emission from the electron transport chain, mtROS surge on both sides of membrane and UCP partial uncoupling (blue pathway). Overload Ca^2+^ influx and excessive mtROS cause Ca^2+^ to leave the mitochondrion via the MPTP and lead to apoptosis (blue dashed pathway).

Beyond their widely recognized roles in energy metabolism, mitochondria play important roles in calcium buffering, cell signaling and cell death. Normally, during glucose oxidation in the cytosol, electrons/reducing equivalents are released and reduced nicotinamide adenine dinucleotide, ATP and protons (H^+^) are produced. In anaerobic glycolysis, each glucose molecule results in the net production of two adenosine triphosphate (ATP) molecules. In aerobic glycolysis, pyruvate enters mitochondria to support Krebs cycle activity and, subsequently, far greater ATP production by the oxidative phosphorylation system is achieved (Fig. 1). This system comprises the ATP synthase, the phosphate carrier, the adenine nucleotide translocator and the electron transport chain, in which electrons provide sufficient free energy to pump protons out of the mitochondrial matrix and into the mitochondrial intermembrane space. This generates a membrane potential Δψ and a proton gradient ΔpH, which together are referred to as *proton motive force*, which in turn is used to drive the activity of ATP synthase. Oxygen is consumed when the electron transport chain is active; as electrons are transferred to oxygen by Complex IV (cytochrome c oxidase) and water is produced.

Ionizing radiation produces radicals, such as the hydrogen radical (H^•^) and hydroxyl radical (OH^•^), from the radiolysis of water as well as reactive oxygen species (ROS) from the irradiation of oxygenated aqueous media. Nevertheless, most ROS in cells, including ROS from low-dose radiation, are thought to originate from mitochondria, due to leakage of electrons from the electron transport chain. It has been estimated that 0.2%–2% of the O_2_ consumed by cells incubated at air saturation (21% O_2_, 160 mmHg, corresponding to ∼203 μmol/L O_2_ solubility in cells at 37°C) form superoxide or other forms of ROS in mitochondria [6, 7]. O_2_ is first reduced to the superoxide anion (^•^O_2_^−^), two of which can then be dismutated by the enzyme superoxide dismutase to hydrogen peroxide (H_2_O_2_). If endogenous ROS production exceeds antioxidative mechanisms, oxidative damage to macromolecules ensues.

Although there are other sources of ROS, e.g. cytoplasmic NADPH oxidase (NOX), unfolding protein response and peroxisomes, mitochondria not only produce most of a cell's ROS, but they may also be more susceptible than the nucleus to their damaging effects. One reason for this susceptibility is that is that the half-lives of ROS and reactive nitrogen species (RNS) in cells are generally very short (≤1 μs), severely limiting their diffusion distances. For example, OH^•^ is most reactive and migrates short distances (1 nm), ^•^O_2_^−^ has moderate range, and H_2_O_2_ can migrate over relatively long distances (1 μm or more). Most mammalian cells, typically 10 – 20 μm in diameter, contain hundreds of mitochondria <1–10 μm long, each with 2–10 copies of mitochondrial DNA (mtDNA). Normal oxidative lesions of DNA, such as the adduct 8-hydroxydeoxyguanosine, have steady state levels 16-fold higher in mtDNA than in nucDNA of rat liver [8]. In addition, mitochondria lack histones, which protect nucDNA from damage, and they have limited DNA repair mechanisms compared with the nucleus [9]. In slowly proliferating tissues, mitochondria have surprisingly rapid turnover rates, characterized by half-lives as short as a few days; and turnover can be accelerated by ionizing radiation [10].

Ionizing radiation causes a transient increase in ROS, from the reduction of oxygen to form ^•^O_2_^−^ and H_2_O_2_, and in RNS, derived from NO. Ward et al [11] noted that low-dose, low-linear energy transfer (LET) radiation produces primary ionization events (electron tracks) and secondary reactive products that are insignificant compared with those produced by endogenous ROS (likely from mitochondria — “mtROS”). A very low-dose gamma-radiation exposure of 1 mGy leads to nucDNA DSBs in only ∼3% of cells, and this effect is proportional to dose [12]. However, cells have highly efficient repair mechanisms, including non-homologous end joining in the G1 phase of the cell cycle and homologous recombination during the S and G2 phases of cell replication. Consequently, cell killing being a stochastic effect, the probability of one DSB (or a pair of breaks) causing nucDNA DSB-related cell killing is unlikely at low doses, as 20–80 DSBs are needed to produce cell death [13].

**Figure 2:**
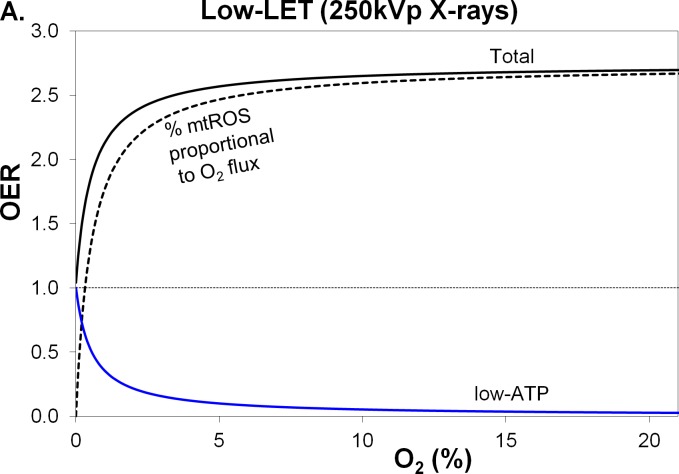

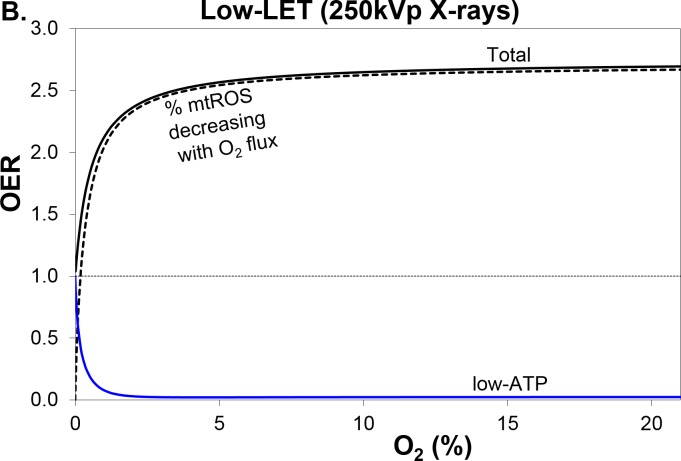
Variation in total OER and its components, with percentage O_2_ in air, for low-LET 250 kVp X-rays The blue line and black-dashed line are the 2 OER components, low-ATP and mtROS, comprising the black line for total OER. MtROS trends shown are **A.** radiation-induced mtROS proportional to O_2_ flux and **B.** fractional mtROS decreasing with O_2_ flux (total OER is unchanged), where mtROS has a more pronounced effect than A. particularly in hypoxic conditions.

Here, we quantitatively analyze the oxygen effect to explain the variation in ionizing radiation's therapeutic or detrimental properties with oxygen level. In the process, we examine and quantify the biological mechanisms underlying the *oxygen enhancement ratio* (OER): the ratio of a chosen measure of radiation-induced damage (*e.g.,* measures including DSBs, chromosomal aberrations and subsequent apoptosis, necrosis and mitotic cell death) when O_2_ is present to that when it is not (Fig. 2A). We demonstrate that damage by both low-LET (*e.g.,* β-, X- or γ-ray exposures) and high-LET (*e.g.,* α-particle) radiations to cells in well-oxygenated tissues is related to the mitochondrial O_2_consumption and production of ROS, and that repair in hypoxic tissues is limited by deficiencies in ATP. In addition, we investigate the hypothesis that the radiation-induced adaptive response of normal cells involves uncoupling of oxidative phosphorylation providing a short-term protective effect. In view of the augmented uncoupling exhibited by some cancers, we speculate that this constitutes a maladaptive response to radiotherapy treatments. Finally, we contend that these analyses, supported by other published studies, provide evidence that, especially for low-LET radiations, the primary radiation targets in oxygenated tissues are mitochondria, which produce ROS that in turn indirectly targets nucDNA.

## RESULTS

### OER variation with O_2_ concentration

Our explanation of the oxygen effect is based on the Howard-Flanders and Alper formula [14] for OER, which can be rearranged as two components (Fig. 2A) for nominal cell parameter values (see Materials and Methods).

1) $$\begin{array}{lll} {\text{OER}}_{O_{2}}^{R} & = & \frac{{\text{OER}}_{\max}^{R}\cdot Co_{2}}{C_{{\text{OER}}_{50}}^{R}+Co_{2}}+\frac{C_{{\text{OER}}_{50}}^{R}}{C_{{\text{OER}}_{50}}^{R}+Co_{2}}\\ & = & \text{mtROS}+\text{low-ATP} \end{array}$$

where OER of radiation R, varies with O_2_ % concentration (*Co_2_*), maximum OER value (*OER^R^_max_*), and half-maximal O_2_ concentration ($C_{{\text{OER}}_{50}}^{R}$).

We define the *mtROS component* of the effect as radiation-induced oxidative damage caused by endogenous mtROS that varies in accordance with hyperbolic increase in O_2_ consumption or flux by mitochondria. The mtROS component shows saturation characteristics similar to those of OER curves when O_2_ levels rise from anoxia (0% O_2_) to severe hypoxia (≤0.1%), hypoxia (≤1%) to normoxia (21% O_2_) [15, 16]. Considering only low-LET exposures in this section, the mtROS component dominates in hypoxia to normoxia (Table 1). When there is greater O_2_ available, the disadvantage conferred by more mtROS is countered by the advantage of greater ATP production capacity.

**Table 1:** Influence of low- and high-LET radiation on OER components and mtROS:O_2_-flux ratio*

Oxygen level (%)	MtROS proportional to O_2_ flux					MtROS decreasing with O_2_ flux				
	mtROS: O_2_-flux ratio	Low-LET		High-LET		mtROS: O_2_-flux ratio	Low-LET		High-LET	
		MtROS (%)	Low-ATP (%)	MtROS (%)	Low-ATP (%)		MtROS (%)	Low-ATP (%)	MtROS (%)	Low-ATP (%)
0	1.00	0	100	0	100	1.99	0	100	0	100
0.1	1.00	33	67	15	85	1.61	54	46	25	75
1.0	1.00	83	17	65	35	1.16	96	4	75	25
10	1.00	98	2	95	5	1.01	99	1	96	4
21	1.00	99	1	97	3	1.00	99	1	97	3

* Designated as unity at 21% O_2_ level for mtROS decreasing with O_2_ flux.

We define the *low-ATP component* as inversely proportional to the O_2_ content (%) and as equal to unity in anoxic conditions, when mtROS levels are zero (Table 1), primarily because of the lack of sufficient O_2_-derived ATP for mitotic DNA repair. The low-ATP component drops rapidly with rising O_2_, making up around a quarter of OER in hypoxic conditions when mtROS increases with O_2_ content.

Radiation damage can be classified as indirect, mainly due to biologic molecules inactivated by radiolysis products of water, and as direct, due to radiation-induced breakage of bonds in macromolecules such as DNA. In the presence of O_2_, radiation-induced mtROS species include highly reactive OH^•^ [17]. Our interpretation of the oxygen effect is that radiolysis products induce mtROS, and this endogenous ROS production by mitochondria is the principal indirect cause of radiation damage observed in nucDNA (e.g., 8-oxo-deoxyguanosine). This explanation aligns with a finding by Chapman et al [18], who employed the OH^•^ scavenger dimethyl sulfoxide and showed that the indirect action of OH^•^ generated by X-rays accounted for ∼30% of the lethal effect on a Chinese hamster fibroblast cell line during anoxia/severe hypoxia (in N_2_), and 62% of cell death under air-saturated conditions.

Two mtROS trends with O_2_ consumption are considered, with the total OER unchanged (Table 1): first, radiation-induced “mtROS proportional to O_2_ flux” (Fig. 2A), and second, “mtROS decreasing with O_2_ flux” (Fig. 2B). The second mtROS trend was evaluated following reports of greater mtROS generation as a percentage of electron flux through the respiration chain in hypoxic conditions [15]. In Fig. 2B, fractional mtROS decreases with O_2_ flux partially based on the data of Hoffman and Brookes [16]. We employ half the mtROS trend modified to produce the typical hyperbolic function for mtROS dependence on O_2_ level. In Table 1 the mtROS:O_2_-flux ratio is designated as unity at the 21% O_2_ level, for mtROS decreasing with O_2_ flux: accordingly, the proportion of mtROS produced under severe hypoxia is virtually twice that at 21% O_2_. Comparing mtROS proportional to O_2_ flux (Fig. 2A) with mtROS decreasing with O_2_ flux (Fig. 2B), the latter exhibits a significant increase in the mtROS component, especially for low-LET exposures in hypoxic conditions.

### Oxygen effect and LET

**Figure 3:**
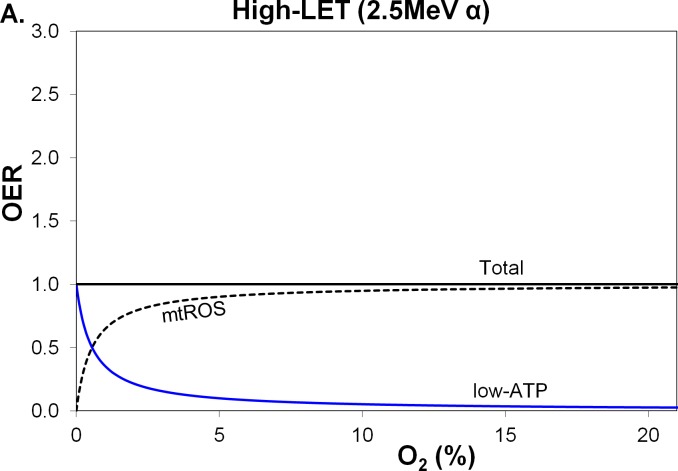

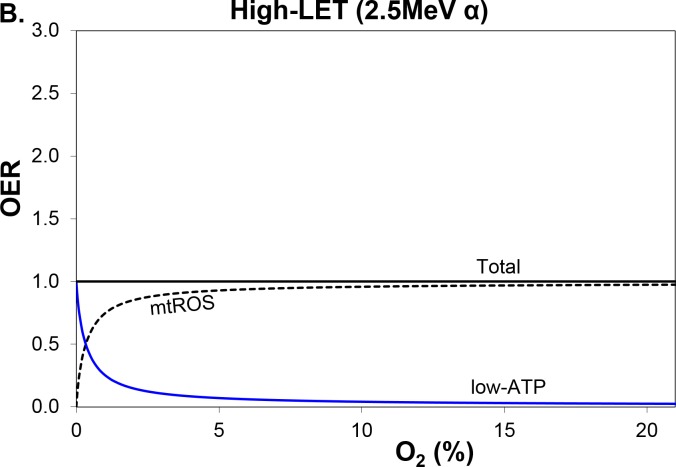
Variation in total OER and its components, with percentage O_2_ in air, for high-LET 2.5 MeV α-particles The blue line and black-dashed line are the 2 OER components, low-ATP and mtROS, comprising the black line for unchanged total OER. MtROS trends appear virtually the same for **A.** radiation-induced mtROS proportional to O_2_ flux and **B.** fractional mtROS decreasing with O_2_ flux (total OER is unchanged).

A high-LET exposure, compared with the same low-LET absorbed dose measured in grays (Gy), has a greater potential to cause tissue damage. The differing characteristics of the oxygen effect for low- and high-LET exposures [19] are assessed by comparing 250 kVp X-rays (LET, 1.3 keV/μm) and 2.5 MeV (LET, 166 keV/μm) α-particles with maximum OER values of 2.74 and 1.00, respectively, and half-maximal O_2_ concentration at 0.55% O_2_ for both. High-LET α-particle tracks produce greater clusters of chemical spurs and radiolysis events in water that lead to the formation of highly oxidising H_2_O_2_ tracks [20]. With increasing LET, the short-range OH^•^ diminish, accompanied by enhanced numbers of long-range ^•^O_2_^−^ and H_2_O_2_ [17]. In the presence of O_2_, while low-LET exposures produce OH^•^ that kill about 60%–90% of mammalian cells, for very high-LET exposures only 25%–30% indirectly succumb [21, 22]. For both low- and high-LET, at 1%–21% O_2_ levels, the mtROS component dominates the low-ATP component (Figs. 2, 3).

Unlike mtROS damage, low-ATP damage is independent of LET. For a particular O_2_ level, the mtROS:low-ATP ratio decreases with higher LET. Consequently, at 1%–21% O_2_ levels, the low-ATP component, due to deficiencies in O_2_-related ATP is two-fold or more for high LET than for lower LET (Table 1). Subsequently, the low-ATP component is greater for high-LET exposures, which reflects the inability to repair more complex DNA damage than that sustained from a low-LET exposure (Table 1).

### Radiotherapy fraction-induced mtROS followed by uncoupling of respiration

The initial contribution to the oxygen effect is from the radiolysis of water, which produces free radicals that stimulate the endoplasmic reticulum to release calcium ions, Ca^2+^. It is our hypothesis that this physiological Ca^2+^ (relatively low Ca^2+^) in turn, enters the mitochondrial matrix and stimulates ATP synthesis [23] as well as the production of mtROS; the latter then triggers a transient, partial uncoupling of oxidative phosphorylation via uncoupling protein 2 (UCP2) [24-26]. To explore the importance of the mitochondrial-centric oxygen effect, we study the biological mechanisms involved in an acute 2 Gy, low-LET exposure under aerobic conditions: allied to a typical curative dose of epithelial tumors being 60–80 Gy in fractions of ∼2 Gy. Specifically, we examine post-irradiation parameters for mitochondria: first, the temporal behavior expected between radiotherapy fractions, based on irradiation studies; and, second, the lower effectiveness of radiotherapy on hypoxic tissue, based on uncoupling studies.

These descriptions are more illustrative than quantitative, since the selected and unmatched parameters are derived from published reports of experiments with exposures of different doses in cultured cells (malignant and non-malignant) and in isolated mitochondria of different organs and species (Table 2). We assume a high-dose (2 Gy), low-LET exposure of non-malignant cells with coupled respiration.

**Table 2:** Estimated percentage difference in mitochondrial-related parameter values before and after a 2 Gy, low-LET exposure under aerobic conditions*

Parameters	Time after exposure	Change post- exposure (%)	Cell and mitochondrial types and reference values
ROS/RNS *vs*. dose	0-3 min	+220% ROS/RNS	3 Gy ^90^Sr β-dose leads to 4.25-fold ↑ ROS gradient – human squamous carcinoma [27]
Δψ *vs*. dose	10 min–1 h	−15% Δψ	4 Gy γ-rays leads to 30% ↓ Δψ – human fibroblasts [28]: similarly, depolarization occurs 3-5 mins [27]
UCP2 *vs*. time	1 h	+16% UCP2	5 Gy leads to 40% ↑ UCP2 – apoptosis-sensitive murine B cell lymphoma [29]
Δψ *vs*. dose	12 h	−7% Δψ	4 Gy γ-rays leads to 14% ↓ Δψ – human fibroblasts [28]
Δψ *vs*. dose	Chronic	−5% Δψ	10 Gy X-rays leads to 26% ↓ Δψ – 3 hamster fibroblast clones [10]
O_2_ consumption *vs*. dose	Chronic	+30% O_2_ consumption	10 Gy X-rays leads to 2.5-fold ↑ O_2_consumption – 3 hamster fibroblast clones [10]

* Experiments utilized cultured cells or isolated mitochondria. Average percentage increase and decrease of cell types indicated by %↑ and ↓, respectively.

Post irradiation-based studies indicate that a high dose of 2 Gy causes an immediate transient increase of radiolysis-induced ROS/RNS levels in a cell that for minutes, is at a ∼3.2-fold higher rate than background levels (Table 2) [27]. This produces a surge in cytosolic Ca^2+^, released by the endoplasmic reticulum (Fig. 1A), and a subsequent uptake of Ca^2+^ into the mitochondrial matrix via the mitochondrial Ca^2+^ uniporter (Fig. 1B). The radiation-induced calcium influx at normal physiological levels into mitochondria also results in an immediate increase in mitochondrial electron transport activity.

Within minutes after an acute exposure, depolarization of the membrane potential occurs (Δψ; ∼ −5 to −15% change), which can last minutes [27], hours [28], or more [10]. Radiation, and similarly increased mtROS, has been shown to trigger UCP2 activity (proton leak uncoupling) and UCP2 expression increases then plateaus beginning 1 hour after exposure (Table 2) [24, 29]. By depressing Δψ, UCP2 acts in a negative feedback loop to decrease mtROS and ATP production for hours or more [25, 26, 30]. This small decrease in Δψ has been observed to revert to nearly normal levels within 12 hours if there is some cell recovery [28], but it can persist if accompanied by genomic instability [10]. Importantly though, an overload of Ca^2+^ influx and excessive mtROS causes mitochondrial permeability transition pore activation and apoptosis (Fig. 1B).

Studies of mitochondrial uncoupling in cells exposed to different O_2_ levels, such as those by Gnaiger et al [15, 31], demonstrate changes in O_2_ consumption, and mtROS production that are dependent on O_2_ levels arising from uncoupling of oxidation phosphorylation in surviving cells [24]. The reciprocal of the half-maximal O_2_ concentration is an important parameter, as it is an index for the O_2_ affinity of the respiratory system. Before radiation exposure, it is assumed that the coupled half-maximal O_2_ value is 0.55% [32] and the maximum O_2_ flow is 40 pmol/s/10^6^ cells. On the basis that a 2 Gy exposure causes total uncoupling, with no change in the respiratory capacity, the half-maximal O_2_ concentration drops to 0.20%, based on data for human endothelial cells [31]. Therefore, in addition to a post-irradiation increase in O_2_ consumption of cells in aerobic conditions [10], compared with coupled respiration, uncoupling causes a more rapid climb in the O_2_ consumption profile (hence mtROS emission) for low O_2_ levels. Specifically, at a low O_2_ level of 0.1% O_2_, radiation-induced uncoupling nearly doubles O_2_ consumption, hence the mtROS component (modelled: fractional mtROS decreases with O_2_ flux, Table 1), which may explain the genetic instability and poorer outcomes sometimes observed after radiotherapy of hypoxic tumors [33].

A known consequence of the uncoupling of oxidative phosphorylation is lower mtROS emissions observed in cells expressing UCP2 [24, 34, 35]. After exposure, this may be accompanied by an increased affinity for O_2_ that, on the one hand, produces more mtROS [10], (especially in hypoxic conditions) [31], yet, on the other hand, provides a beneficial radiotherapeutic environment for well-oxygenated non-malignant cells to repair the sub-lethal damage that usually occurs within 24 hours of exposure. Similarly, uncoupling in malignantly transformed cells has been observed for moderate doses (0.4 and 0.8 Gy) and high doses (9 and 10 Gy) of X- and α-radiation, evidenced by modified oxidation phosphorylation activity, cytochrome *c* oxidase and O_2_ consumption per mtDNA copy number [36, 37]. Likewise, Voehringer et al. [29] showed radiation-induced UCP2 activation lead to mitochondrial uncoupling and even cell death, in an irradiated murine B cell lymphoma model, although the hypothesized radiation-induced loss of membrane potential was observed by others [27, 28]. Even though radiation-induced uncoupling has been demonstrated under particular cellular conditions [38], there is plainly a need to experimentally verify in the one study that radiation exposures result in UCP2 activation plus a loss of membrane potential. Albeit, even allowing for the diverse and somewhat incompatible nature of the studies reported, this analysis suggests that uncoupled respiration is an important mechanism involved in sub-lethal and lethal radiation exposures of both malignant and non-malignant cells.

## DISCUSSION

### Factors influencing radiation-induced mtROS emission

**Figure 4:**
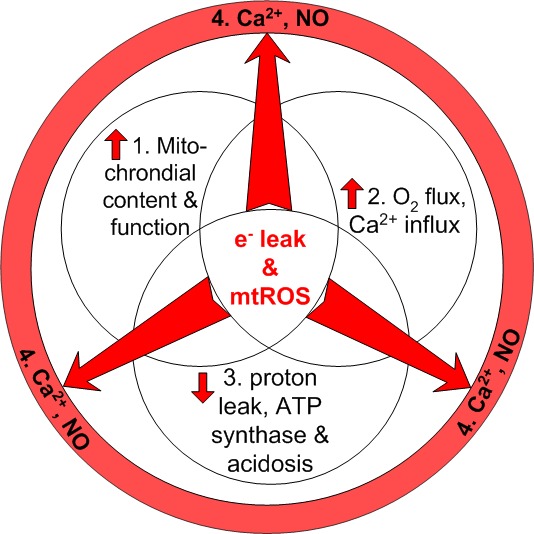
Mitochondrial factors modified by irradiation influence mtROS emission The first three factors are endogenous: greater mitochondrial content and function, greater O_2_ consumption and Ca^2+^ influx, and decreased proton leak, ATP synthase and acidosis. These factors are accompanied by greater electron leakage and resulting mtROS generation. The fourth factor is exogenous, illustrating the egress of Ca^2+^ and NO influencing the mtROS emission in bystander cells.

We propose a major role for mitochondria in radiation-induced cell death and the oxygen effect. While the exact nature of the processes involved are not fully understood, it is useful to consider the known factors that affect radiation-induced mtROS emission. Two factors that increase mtROS are a higher number of dysfunctional mitochondria in a cell, and a greater local O_2_ concentration. A third factor that decreases mtROS emission is proton leak, or the uncoupling of electron transport chain activity from ATP synthase activity (Fig. 4). A fourth factor is that mtROS can be amplified by the generation and migration of NO and Ca^2+^, impinging upon neighboring mitochondria and cells.

#### Higher mitochondrial content and functional capacity

Mitochondrial mass in peripheral lymphocytes increased 2.3-fold after a 4.5 Gy low-LET radiation dose, and increased by a larger factor after a 9 Gy dose, in patients undergoing total body irradiation therapy [39]. Similarly, fractional increases in mitochondrial fusion or mitochondrial mass have been seen after lower doses of low-LET and high-LET radiation to neurons and lung tumor cells [36, 40]. In an effort to aid repair after irradiation, a cell acquires a greater capacity to produce ATP, offset by mitochondrial dysfunction denoted by more mtROS production [10, 38].

#### Greater O_2_ consumption that coincides with ATP demand

Howard-Flanders and Alper [14] first quantified OER using gram-negative *E. coli* bacteria. This OER formulation is also applicable to malignant and non-malignant human cells (eukaryotic cells); relevant here is the fact that mitochondria have gram-negative bacterial origins, and first invaded cells to establish an endosymbiotic relationship. Although there are many influences (*e.g.,* membrane potential) on mtROS emission, we model the mtROS generated as proportional to, or fractional decrease with, O_2_ consumption, showing saturation kinetic characteristics, rather than a linear regression, with O_2_ tension. Mitochondrial-derived ROS are produced on both the matrix side and cytoplasmic side of the mitochondrial inner membrane, which is impermeable to ^•^O_2_^−^ but not H_2_O_2_. (Fig. 1B) [7]. MtROS O_2_-dependent trends also depend on other issues, such as metabolic conditions, antioxidant activity, mitochondrial site of ROS origin and drop in phosphorylation efficiency during ADP limitation at high O_2_ levels [6, 16, 41]. The mitochondrial sites of ROS generation by irradiation are comparatively uncharacterized and may involve pro-apoptotic, enzymatic events requiring cytochrome *c* release [17, 42]. Mitochondria usually have a high reserve capacity to produce ATP in times of need, such as when cells are irradiated. Stable clones of hamster fibroblasts increased their O_2_ consumption two-fold after being subjected to 10 Gy low-LET X-rays [10]. A similar elevation in O_2_ consumption was measured for a lower dose (0.8 Gy) of high-LET α-radiation [33].

#### Proton leak uncoupling of the electron transport chain from ATP synthase activity

Mitochondrial uncoupling, through proton leaks, lowers both ROS emission and the efficiency of mitochondrial ATP production [25, 31]. There are two general classes of proton leak: a basal leak and an inducible form that employs uncoupling proteins and other mitochondrial inner membrane proteins, including the ADP/ATP translocator [43]. The acute activation mechanisms of the uncoupling proteins involve ROS, ROS byproducts (*e.g.,* 4-HNE) and protein post-translational mechanisms, notably glutathionylation [25]. Superoxide was shown to stimulate UCP1, 2, 3 activities in the presence of fatty acids [26]; likewise, high-dose γ-rays stimulate UCP2 activity, which thus lowers mitochondrial membrane potential [24]. Consequently, UCPs may increase radioresistance by lowering membrane potential and thereby minimizing ROS emission [24-26].

#### MtROS can be amplified by the generation and migration of radiolysis-initiated ROS, Ca^2+^ and NO

Subjecting cells, including tumor cells, to 1% NO in an inert gas has a sensitizing effect on radiation-induced cell death, similar to that of oxygenation [44]. Our analysis of the oxygen effect accommodates this fundamental finding. In both O_2_ and NO atmospheres, mitochondria play an important role in the radiation effects on cells [27]. Of particular pertinence to the biological mechanisms underlying the oxygen effect, the *HIF1α* gene is regulated not only by O_2_, but also by NO and ROS [45]. This is especially relevant at low O_2_ levels, at which the low-ATP component dominates. Support for the mtROS OER component includes the finding that, in the presence of O_2_, the radiation activation of mitochondrial inducible NO synthases and the radiolysis of water are major sources of ROS/RNS, with higher LET being more likely to generate long-range radicals capable of reaching nucDNA [17, 46].

Particularly at high doses, severe radiation damage of mitochondria results in mutated mtDNA, catastrophic mitochondrial Ca^2+^ influx, the release of cytochrome *c*, and the onset of an irreversible mitochondrial permeability transition associated with apoptosis and necrosis. An initial ionizing event can be amplified by Ca^2+^ signaling [27] across the cytoplasm to other mitochondria of the affected cell or even to bystander cells [17, 38, 47]. While further research is clearly needed, we have examined whether low-LET-generated Ca^2+^ and mtROS are more signal-related than overwhelmingly detrimental, and whether the converse is true for high-LET clustered ionizations [48]. Our initial conclusion is that this OER analysis better relates to low-LET than to the unrepairable damage of high-LET exposures.

### Oxygen effect and dose

While the relationships of cell survival curves to dose are beyond the scope of this analysis, experimental studies report differences in the cellular effects of low- and high-dose exposures for both low- and high-LET radiation [49]. No analysis of the differing effects of low-dose or high-dose on OER curves has been pursued, although the review of Wenzl and Wilkens [49] noted variable OER behavior. Indeed, OER values can increase, decrease or remain nearly constant with increasing radiation doses, depending on the cell line and radiation exposure used.

Notwithstanding, our mitochondrial-related analysis has a bearing on the debate over the origin of the “shoulder” region of survival curves, especially evident in doses of <10 Gy of low-LET radiation under aerobic conditions. This shoulder region is exploited in split dose or multi-fractionated radiotherapy regimes with intervals, leading to a decreased response to a second exposure of aerated tissue. On the basis of our analysis, there is clearly a need to experimentally explore whether radiation-induced UCP2 activates uncoupling of the oxidative phosphorylation system (proton leak) in mitochondria. In particular, where a first radiation exposure and uncoupling modifies the oxidative stress generated by a second exposure and its subsequence relevance to fractionated/chronic exposures and to radiation adaptive responses.

One explanation for the success of fractionated therapy is that hypoxic tumor cells have time to reoxygenate after apoptotic cells have been removed following the initial exposure. There are difficulties in explaining the shoulder region in terms of the DSB dose response, which is generally linear for a very wide range of doses [50]. However, the shoulder region of cell survival curves is usually interpreted in one of two ways. First, dose-dependent detrimental effects to cells may eventually lead to lethal damage at high dose. Second, enzymatic repair, aided by low dose promoting greater mitochondrial fusion and mitochondrial mass [40], becomes ineffective at high dose.

Repair of sub-lethal radiation damage occurs preferentially in different phases of the cell cycle, with checkpoints that monitor ATP before allowing the cell cycle to progress [51]. Especially relevant to the low-ATP OER component is that mitotic cell death after irradiation is more prevalent in hypoxia, when ATP is in short supply. This mitotic cell death pre-empts DNA repair by mitotic homologous recombination.

The multi-hit cell damage of X-irradiation at high dose (4 Gy) and dose rates (3.3 Gy/min) compromise mitochondrial maintenance and regeneration, as mitochondrial protein import decreases in human fibroblasts [28], whereas at low dose (0.1 Gy) and dose rates (2 mGy/h), membrane potential was increased and protein import was found to be enhanced compared to controls. This appears to have a knock-on effect: in one study in rat splenocytes, low-dose exposure (∼0.01–0.1 Gy) demonstrated a transitory inhibition of mitosis followed by an increase in cell division, likely linked to sub-lethal repair [52]. At higher doses, cell proliferation was reduced.

Assuming a linear dose dependency, for relatively low-dose, low-LET exposures, mtROS production would be close to basal levels (Table 2). This may imply that the mtROS component of OER curves is more closely related to the controlled progression of Ca^­2+^-initiated signaling [48], cell cycle arrest and apoptosis than to an immediate, catastrophic mitochondrial permeability transition, large depolarization and release of cytochrome *c* into the cytosol. Therefore, based on the mtROS component having the dual traits of oxidative damage and greater ATP production, we suggest, particularly for low-dose low-LET exposures, that the shoulder region should not be interpreted as exclusively damage- or repair-related, but as a combination of these two mechanisms.

### Radiosensitivity varies with supplies of O_2_, glucose and ATP

Levels of anaerobic glycolysis *versus* aerobic oxidative phosphorylation seesaw, depending on the availability of O_2_, nutrients and other factors. Oxygen tension has a strong influence on wound healing, where epithelial growth is proportional to O_2_ tension up to normoxic and even higher levels, although cell replication is rare at <2.6% O_2_ and virtually non-existent at <0.7% O_2_ [53]. Hypoxia inducible factors (HIFs), a small group of master transcription factors have central roles in the adaptation to hypoxia, perhaps even up to levels of 20% O_2_ [54]. Under normal O_2_ conditions, when the mtROS OER component dominates, glycolytic metabolism is inhibited at the expense of oxidative phosphorylation activity. By contrast, in hypoxia, when the low-ATP OER component dominates, there is increased protein unfolding, yet decreased protein turnover, ATP demand and oxidative stress. Cellular mitochondrial content is reduced in anaerobic compared with aerobic cultures, measured by citrate synthase activity in human primary fibroblasts, which supports a lesser role for mitochondrial damage in low O_2_ conditions [55]. The shift from oxidative phosphorylation to anaerobic glycolysis is accompanied by a far lower ATP production capacity and an energy deficit, which affects a cell's ability to repair and replicate after stress, including that from radiation.

Of the myriad tumor driver genes, only the high-frequency mutations in the p53 tumor suppressor gene are significantly associated with rising cancer incidence rates in aging adults [56]. It is well known that stresses such as ionizing radiation increase nuclear p53 protein levels as a consequence of nucDNA damage and that therapy-level exposure can accelerate the processes of aging and cancer [57]. Deficiency in the p53 protein confers not only apoptotic evasion, but also a preference for a glycolytic metabolism [58]. Nevertheless, most of the ATP in tumor cells is thought to be produced through glutamine-driven oxidative phosphorylation, in both normoxic and low O_2_ conditions [59]. Cells have evolved coping strategies at low O_2_ levels such as increasing p53 activation of a G1-phase cell cycle checkpoint distinct from nucDNA damage pathways [60]. In addition, hypoxia induces anaerobic glycolysis, and the consequential increase in lactate production and secondary effects (Fig. 4), such as extra-mitochondrial, extra-cellular acidosis. These may bring about a greater proton burden, loss of membrane potential and mitochondrial remodeling, minimizing the effects of radiotherapy [61].

Tumor cells generate increased mtROS, partially as a result of mitochondrial dysfunction [33]. When irradiated, some tumor cells have higher levels of the uncoupling protein UCP2 and mitochondrial proton leak, thereby lessening mtROS and conferring resistance to cytotoxic treatments. This was shown in a study by one of us [24], which also found that after a low-LET dose of 20 Gy in aerated conditions, resistant subclones of leukemia cells have elevated UCP2 activity, suppressing ROS and subsequent nucDNA damage as measured by 8-oxo-deoxyguanosine. These resistant subclones also augment their metabolism, utilizing fatty acids when glucose levels are low. Conversely, as illustrated earlier, hypoxic tumors that exhibit radiation-induced uncoupling can also be expected to exhibit enhanced mtROS generation. Similarly, inhibition of UCP2 increases mtROS and restores sensitivity to some chemotherapeutics [62]. These studies showed that uncoupling is an important aspect governing how cells adapt to a radiation exposure. It would be worth confirming in future studies the premise that a tumor's radioresistance is due in part to an extended period of adaptive uncoupling.

### Are radiation effects mitochondria-centric rather than nucleus-centric?

Published experimental research, and this analysis of the radiation oxygen effect, identify a major role for uncoupled respiration in mitochondria. They also provide strong, if not definitive, evidence for the dominance of indirect actions of radiation involving mitochondria over direct actions on the nucleus. For example, both low-LET and high-LET irradiation of fibroblasts and other cells can produce mitochondrial dysfunction accompanied by persistent ROS production and genomic instability [10, 17, 33, 46, 63]. While many would argue that nucDNA DSBs are a crucial facet of radiation damage to cells, Franken et al [64] showed that the relative biological effectiveness (RBE), based on the number of DSBs, remains about unity, no matter the level of LET, whereas chromosomal aberrations, lethal damage and reproductive death are much more LET-dependent.

Experimental evidence, acquired mainly after exposures with charged particle microbeams or radiolabels with short-range impact, shows that direct damage to the nucleus can be the most effective cause of radiation effects such as DNA damage and cell killing. Yet irradiation of the cytoplasm or mitochondria indirectly causes these same effects, sometimes with lower efficiencies [65]. For example, Tartier et al [47] irradiated cytoplasm with high-LET helium ions and showed that direct hits to the nucDNA of HeLa cells were not needed to cause damage, as measured in the form of p53 binding protein 1 (53BP1) foci at DSB sites in irradiated and bystander cells. The study showed that the damage was caused indirectly, via production of ROS and RNS.

Furthermore, Zhang and colleagues [46] induced DNA oxidative damage and DSBs by targeting the cytoplasm of wild type ρ^+^ (mitochondria-endowed) human small airway epithelial cells with a helium ion microbeam of 1 μm width. The findings were compared to those from mtDNA depleted (ρ^0^) cells. A significant increase in autophagy and micronuclei indicated that genomic instability had resulted in the ρ^+^ cells. These studies, conducted in oxygenated environments, increasingly point to mitochondria as essential in mediating cytoplasmic radiation-induced genotoxic damage in targeted or bystander mammalian cells.

Conversely, some studies support the view that lower O_2_ levels tend to more nucleus-centric damage. Low-LET ^137^Cs γ-exposures (0–10 Gy) under chronically hypoxic conditions have been shown to increase G1-associated nucDNA DSBs, leading to greater genetic instability and lower clonogenic survival [66]. The selection of the nucleus as the primary cellular radiation target is supported by the observing that when the Auger-emitter ^125^I is incorporated in close proximity to the nucleus it is much more effective at producing DSBs or cell death than ^125^I sited in the cytoplasm [67, 68].

Lastly, Leach et al [27] observed radiation-induced ROS/RNS in osteosarcoma ρ^+^ cells, but not in mitochondria-depleted ρ^0^ cells. There are reports that both normal and malignant irradiated ρ^0^ cells are more radioresistant than ρ^+^ cells in the presence of O_2_ [69, 70]. Yet in hypoxic conditions, there was little difference in the radiosensitivity of ρ^+^ and ρ^0^ cells. This would be expected in light of our hypothesis that the oxygen effect can be quantitatively explained by OER comprising two components, the most prominent being the generation of mtROS under aerobic conditions. Experimental studies are needed for confirmation of this hypothesis.

In conclusion, we acknowledge that radiation damage to the cell nucleus is of ultimate importance. However we propose that this nuclear damage generally arises indirectly as a knock-on effect, via Ca^2+^ flux and damage to mitochondria, which appears to be the most likely initiator of radiation-induced ROS, nucDNA damage, cell death and perhaps oncogenesis. Consequently, we suggest that further research in radiotherapy would benefit from greater emphasis on aspects involving mitochondria, the contribution of the various sources of ROS, and uncoupled oxidative phosphorylation.

## MATERIALS AND METHODS

The analysis utilizes the OER, which varies from unity in anoxia to typically about three for low-LET radiation, with radiosensitivity varying most rapidly with O_2_ partial pressures below ∼2% atmospheric (Fig. 2A). Areas in solid tumors can be anoxic, while in normal cells physiological O_2_ tension varies widely, ranging from ∼11% concentration in the blood of large arteries to as low as 0.1% within cells [16].

We based our analytical methods on a competitive type of reaction expression developed by Howard-Flanders and Alper [14] for OER of radiation R and its variation with O_2_ % concentration (Co_2_)(Fig. 2A), or O_2_ pressure in air:

2) $${\text{OER}}_{O_{2}}^{R}=\frac{{\text{OER}}_{\max}^{R}\cdot Co_{2}}{C_{{\text{OER}}_{50}}^{R}+Co_{2}}+\frac{C_{{\text{OER}}_{50}}^{R}}{C_{{\text{OER}}_{50}}^{R}+Co_{2}}$$

where the maximum OER value, (*OER^R^_max_*) is obtained in the presence of 100% O_2_ (one atmosphere is 101.3 kPa, 760 mmHg or 760 torr). The value of (*OER^R^_max_*) is very similar to that for the OER for normoxia (${\text{OER}}_{21\%O_{2}}^{R}$)) conditions (Table 1). This analysis utilized ${\text{OER}}_{21\%O_{2}}^{R}$ values for the lowest and highest of nine radiation types with increasing LET derived by Barendsen et al [19]. Half-maximal O_2_ concentration ($C_{OER_{50}}^{R}$) is the percentage O_2_ for which the cellular radiosensitivity is midway between the anoxic and fully oxygenated responses. A value of 0.55% O_2_ was assumed for both low- and high-LET radiation; to our knowledge there is no discernible trend in $C_{OER_{50}}^{R}$ with LET [32].

The O_2_ flux variation with *Co_2_* in mitochondria, controlled by several enzymes, can be represented by a rectangular hyperbolic function [15]:

3) $$Jo_{2}=\frac{J_{\max\cdot }Co_{2}}{C_{J_{50}}+Co_{2}}$$

Assuming that radiation-induced oxidative stress (an abnormal state) is primarily linked to the leakage of electrons by the mitochondrial electron transport chain, proportional to O_2_ consumption *Jo_2_* (pmol/s /cm^3^), Eqn. 2 can be rearranged into two components as shown in Eqn. 1. The first OER component mtROS is similar in form to Eqn. 3. The second OER component, low-ATP is unity for cells in anoxia and diminishes with the O_2_ tension. (This does not account for uncoupled respiration under hypoxia; see Results.).
